# The Feasibility of One-Stage Instance Segmentation for Detecting Oral Potentially Malignant Disorders in White-Light Clinical Photographs: A Proof-of-Concept Study

**DOI:** 10.3390/dj14080462

**Published:** 2026-07-23

**Authors:** Swee Ling Low, Hui Teng Chong, Jin Wen Liew, Spoorthi Ravi Banavar, Prashanthi Chippagiri, Elaine Wan Ling Chan, Wan Siti Halimatul Munirah Wan Ahmad, Suan Phaik Khoo

**Affiliations:** 1 Division of Clinical Oral Health Sciences, School of Dentistry, IMU University, Kuala Lumpur 57000, Malaysia00000034441@student.imu.edu.my (H.T.C.); suanphaik_khoo@imu.edu.my (S.P.K.); 2 Department of Data Science and Artificial Intelligence, School of Computing and Artificial Intelligence, Faculty of Engineering and Technology, Sunway University, Kuala Lumpur 47500, Malaysia; elainecwl@sunway.edu.my (E.W.L.C.);

**Keywords:** oral potentially malignant disorders, deep learning, convolutional neural network, instance segmentation, early detection

## Abstract

**Objectives**: Oral potentially malignant disorders (OPMDs) carry a variable risk of malignant transformation, making early detection important. Deep learning relies on specialized imaging, which is often inaccessible in routine practice, and detection from standard clinical photographs remains poorly characterized. We assessed the feasibility of automated OPMD detection from white-light intraoral photographs, compared two convolutional neural network paradigms (global classification with DenseNet-121 versus one-stage instance segmentation with YOLOv8), and identified the main barriers to clinical translation. **Methods**: A dataset of 1500 photographs (750 OPMD and 750 non-OPMD) from institutional archives and publicly accessible sources was split in an 80:20 ratio for training and testing. Lesion boundaries were annotated by three trainees using Cytomine and validated by three specialists. The DenseNet-121 and YOLOv8-large-segmentation models were evaluated for accuracy, sensitivity, specificity, precision, F1 score, and Wilson 95% CI. **Results**: DenseNet-121 required extensive manual lesion cropping to converge, negating the automation rationale. YOLOv8-large-segmentation reached 62.2% accuracy (95% CI 56.4 to 67.6), 75% sensitivity (95% CI 67.3 to 81.4), 59.7% precision (95% CI 52.4 to 66.5), 49.3% specificity (95% CI 41.3 to 57.4), and an F1 score of 66.5%, detecting lesions in 96% of the test set. High sensitivity was obtained at a low confidence threshold of 0.1, with correspondingly reduced specificity, and the model produced interpretable color-coded segmentation masks. **Conclusions**: One-stage instance segmentation is the more viable direction and yields spatially interpretable output, but performance at this dataset scale is not yet clinically sufficient. Dataset scale, threshold calibration, low specificity, and absent patient metadata are the key barriers to address.

## 1. Introduction

Oral squamous cell carcinoma (OSCC) is the most prevalent malignancy of the head and neck region and accounts for approximately 90% of all oral cancers [1,2]. According to GLOBOCAN 2020, there are an estimated 377,713 new cases and 177,757 deaths attributed to lip and oral cavity cancer each year worldwide [3]. A disproportionately high burden falls on low- and middle-income countries (LMICs), where nearly two-thirds of OSCC cases occur [4]. Within Southeast Asia, Malaysia reports one of the highest incidence rates at 4.12 cases per 100,000 population [5]. Despite advances in oncological management, survival outcomes for OSCC remain poor, largely because of late-stage diagnosis at presentation [6,7,8]. A key opportunity for improving outcomes lies in the early identification of OPMDs, a heterogeneous group of lesions that carry a variable risk of malignant transformation into OSCC. The systematic identification and monitoring of these conditions through population-based screening have been recognized as an important strategy for earlier diagnosis and better treatment outcomes [9]. OPMDs comprise a clinically heterogeneous group of entities including leukoplakia, erythroplakia, oral submucous fibrosis, oral lichen planus, and proliferative verrucous leukoplakia, whose malignant transformation rates vary widely, from approximately 3.5% for leukoplakia to substantially higher figures for other entities, underscoring the importance of their early and reliable identification [10].

Current oral cancer screening programs face substantial challenges. Primary healthcare providers often lack adequate training to reliably identify OPMDs, with a reported diagnostic sensitivity of approximately 57.8% and specificity between 31% and 53% [11,12]. The diverse and overlapping clinical presentations of oral lesions complicate consistent identification and contribute to delayed specialist referral. Adjunctive techniques such as vital staining, autofluorescence, and chemiluminescence are available but were found to be insufficient for routine diagnostic triage by the 2017 American Dental Association clinical guidelines [13]. Histopathological examination remains the gold standard for the definitive diagnosis of OPMDs. Its invasiveness, resource intensity, and limited accessibility in low-resource settings make it impractical for widespread population screening [14]. Therefore, there is a clear need for non-invasive, cost-effective, and accessible screening tools.

Developments in computer vision and deep learning present promising opportunities for addressing this unmet clinical need. Automated image analysis systems could provide real-time decision support to clinicians, support patient self-examination, and enable teleconsultation-based referral pathways. This is particularly relevant in LMICs with limited specialist access. A study by Gomes et al. [15] demonstrated substantial agreement between in-person specialist evaluations and those derived from mobile device-captured oral photographs, which supports the feasibility of image-based remote assessment.

Among deep learning approaches, convolutional neural networks (CNNs) have become the dominant architecture for photographic image analysis, using hierarchical feature extraction through convolution, pooling, and fully connected layers [15]. Studies have shown the effectiveness of CNN architectures such as GoogLeNet, AlexNet, ResNet, and VGGNet in classifying precancerous skin lesions [16]. CNN-based laryngeal tumor detection in laryngoscopy images has shown accuracy comparable to that of experienced clinicians [17]. Despite these developments, the application of CNNs to OPMD classification using standard white-light clinical photographs remains underexplored, with most published work focused on specialized imaging modalities that are not routinely available in clinical practice [18,19]. Within oncology specifically, deep learning classification and detection models have shown early promise on oral lesion images, but most have been evaluated on curated or single-source datasets, and few have assessed instance segmentation on unprocessed white-light photographs, leaving the reproducibility and generalizability of such pipelines insufficiently characterized [20,21].

**Figure 1:**
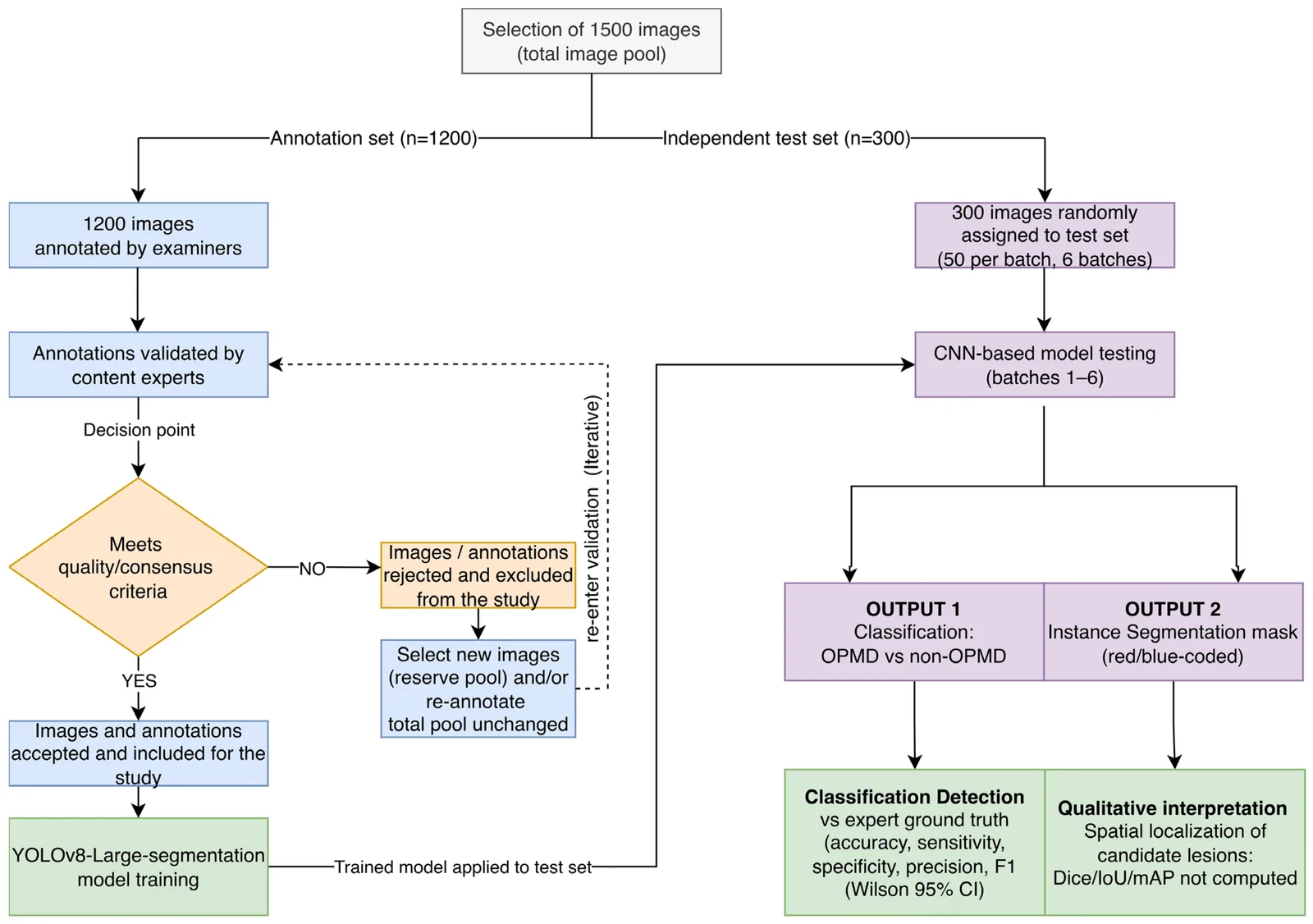
Process flow of image selection, image annotation, model training, model testing, and automated mask generation for each batch of images. Solid arrows indicate the sequential workflow, whereas the dashed arrow indicates the iterative re-validation and re-annotation process.

Preliminary studies using architectures including U-Net, YOLOv5, ResNet-152, DenseNet-161, Inception-v4, and several EfficientNet variants have begun to show feasibility for OPMD classification and detection using clinical photographs [20,21], but a systematic evaluation of purpose-built detection and segmentation pipelines is still lacking. DenseNet architectures are characterized by densely connected inter-layer pathways that improve feature reuse and gradient propagation [22,23]. YOLOv8 represents one of the best approaches in one-stage object detection and instance segmentation, and it enables lesion localization, classification, and boundary delineation in a single network forward pass [24]. CNN architectures process input images through sequential convolution and pooling layers for hierarchical feature extraction, after which a fully connected layer generates the final classification output (Figure 1) [16].

This study aimed to assess the feasibility of binary OPMD or non-OPMD detection from standard white-light intraoral photographs, to determine whether a global classification paradigm (DenseNet-121) or a one-stage instance segmentation paradigm (YOLOv8-large) is the more viable architectural direction for this localized task, and to characterize the main performance and translational barriers, including dataset scale, decision threshold calibration, and the absence of patient-level metadata, so as to define an evidence-based agenda to establish a direction and to quantify the obstacles to it.

## 2. Materials and Methods

### 2.1. Study Design Overview

This study used a retrospective design to train and evaluate two CNN-based deep learning architectures for the automated binary classification and segmentation of intraoral clinical photographs. Images were collected in batches of six, each comprising 250 images. The dataset was randomly split 80 to 20 into training and testing partitions. Lesion boundaries in training images were annotated and specialist-validated before model training. The pipeline produced two outputs, a binary classification (OPMD versus non-OPMD) and a segmentation mask delineating the lesion boundary. Ethical approval was obtained from the institution’s Research Ethics Committee. Written informed consent was obtained from all patients whose archived images were used.

### 2.2. Sample Size Calculation

A systematic review by Richards [25], involving 22 epidemiological surveys, estimated the global prevalence of OPMD to be 4.47%. Napier and Speight [26], in their review of the literature, reported that the global prevalence of OPMD ranges between 1% and 5%. For disease prevalence estimation, Buderer [27] proposed a statistical method to calculate sample size requirements based on hypothesized values of sensitivity and specificity, a clinically acceptable level of precision, and the estimated prevalence of a disease. A sample size calculator applying this method was used to determine the requirement for this research (Arifin [28]). To achieve 90% sensitivity, 90% specificity, 95% confidence, and a 10% margin of error for detecting OPMD in a sample set with an OPMD-to-non-OPMD ratio of 1 to 1, and allowing for a 10% dropout rate, a minimum of 385 samples each for OPMD and non-OPMD was required. This calculation follows diagnostic accuracy principles and ensures adequate precision for estimating sensitivity and specificity; it does not address the distinct requirements of deep learning model development, in which dataset scale relative to model complexity and external validation are the primary determinants of performance, as discussed in Section 4.5 and Section 4.6.

### 2.3. Dataset

A total of 1500 colored intraoral clinical photographs were collected and labeled into two classes, with 750 images representing OPMD and 750 images representing non-OPMD. These photographs were collected in batches of six, each comprising 250 images (125 images per class), sourced from the university’s archives (written informed consent was obtained from all patients) and from various publicly accessible platforms using search engines. The OPMD and non-OPMD conditions included are listed in Table 1.

The lesions and conditions included in the OPMD class are common, especially in the South and Southeast Asia regions [10,29,30]. The non-OPMD category included oral lesions that closely resemble OPMD and therefore serve as differential diagnoses in clinical practice. OSCC was also included in the non-OPMD category, given its importance as a key differential diagnosis of OPMD. This decision was made to improve the clinical applicability of the dataset and to avoid excluding relevant cases. The OPMD and non-OPMD entities were defined according to the current WHO classification of oral potentially malignant disorders (2020/2021 international consensus); conditions not classified as OPMD under this consensus, such as chronic hyperplastic candidiasis, were assigned to the non-OPMD category.

To support more accurate and higher-quality results, selected images were kept at a minimum resolution of 72 dots per inch (dpi). The dataset varied in image quality across angle, light exposure, sharpness, and zoom. The regions surrounding lesions, such as the teeth, some facial features (for example, chin, lips, and cheeks), the mouth mirror, and the retractor, were included. To reduce the chance of errors, images with distracting elements, such as a busy background containing non-relevant objects, were excluded. The final dataset was divided randomly and in a stratified manner so that the proportional representation of each class remained consistent with the original dataset, with 80% allocated for model training and 20% for model testing. This meant that from 250 images from each batch, 200 of them were used for training and 50 for testing. The result was a total of 1200 images assigned to training and 300 images set aside for testing. A summary of the workflow is shown in Figure 1. Images from both the institutional archive and the publicly accessible online sources were subject to the same inclusion, resolution and exclusion criteria and to the same specialist validation; the residual risk of near-duplicate images is addressed in Section 4.5.

### 2.4. Image Annotation

**Figure 2:**
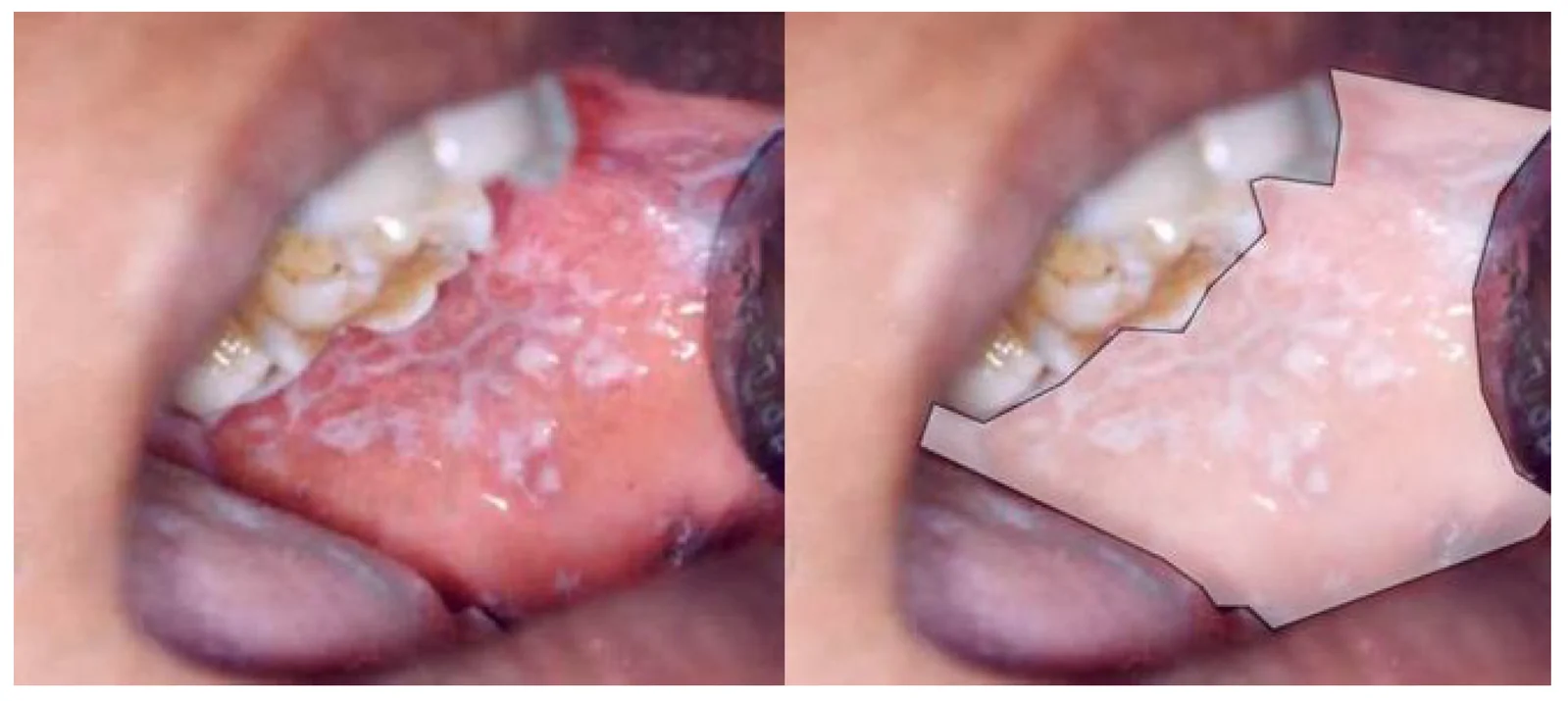
A representative example. (**Left**), an image of oral lichen planus on the left buccal mucosa. (**Right**), the annotation of the lesion, excluding areas such as teeth and the mouth mirror.

Image annotation was performed on the Cytomine collaborative digital pathology platform [31], a web-based open-source tool designed for large-scale image annotation and analysis. Before annotating the dataset, three non-specialist examiners/annotators (LSL, CHT, LJW) were trained on image annotation by three Oral Medicine and Pathology specialists (SPK, SRB, PRC). Post training, the training dataset of 1200 images was then annotated equally and independently by the three annotators. Lesions were annotated by delineating their boundaries using a polygon shape while excluding areas such as teeth, the mouth mirror, or cheek retractors and were labeled according to their class, either OPMD or non-OPMD (Figure 2). The annotations were validated by three specialists to confirm accurate annotation for model training. Annotations judged inaccurate were returned for revision before re-submission for specialist re-validation (Figure 1).

### 2.5. Model Architecture and Training

Two types of CNN-based deep learning models were used in this study. DenseNet-121 was used for image classification, and YOLOv8-large-segmentation was used for one-stage detection and segmentation. Among the DenseNet pretrained variants, DenseNet-121 is the lightest model without a marked loss of accuracy.

YOLOv8 [24] is one of the best one-stage detection and instance segmentation frameworks, with segmentation capability that highlights the focus area of the detected regions. YOLO models come in different variants, typically denoted n (nano), s (small), m (medium), l (large), and x (extra-large), which represent different model sizes and complexities. These variants allow for a balance between speed and accuracy based on computational resources and application requirements. In this study, the YOLOv8-large-segmentation model was used.

For DenseNet-121, three training settings were carried out, and the results were compared. Training A used uncropped images classified into seven groups (erythroplakia, leukoplakia, PVL, OSMF, OLL, OLP, and non-OPMD). Training B used manually cropped images focused only on the lesion, also classified into the same seven groups. Training C used similar cropped images classified into only two groups (OPMD and non-OPMD). The DenseNet-121 classifier was implemented in PyTorch 2.1x using the torchvision library and initialized with ImageNet-pretrained weights; input images were resized to 224 × 224 pixels and models were trained for 100 epochs, with stochastic gradient descent and Adam optimizers compared across runs. All other training hyperparameters (learning rate, batch size, weight decay, and data augmentation) were left at their framework defaults, and no bespoke hyperparameter tuning was performed.

In the YOLOv8-large-segmentation model, instance segmentation features were applied using the validated annotated regions. To extract the annotations, analyses were launched from the Cytomine platform to generate comma-separated values (CSV) files containing the coordinates of annotated polygons. These files were pre-processed to comply with the YOLO input format, which enabled the model to learn from the annotated regions of the lesions for prediction. A confidence threshold of 0.1 was applied during inference to favor detection sensitivity. The YOLOv8-large-segmentation model was implemented using the Ultralytics framework and fine-tuned from COCO-pretrained weights on the annotated dataset. Training was performed using the default hyperparameters of the Ultralytics YOLOv8 (v8.0.0) framework (default optimizer, learning rate, batch size, input image size, and data augmentation schedule), without bespoke hyperparameter tuning.

### 2.6. Model Testing

**Figure 3:**
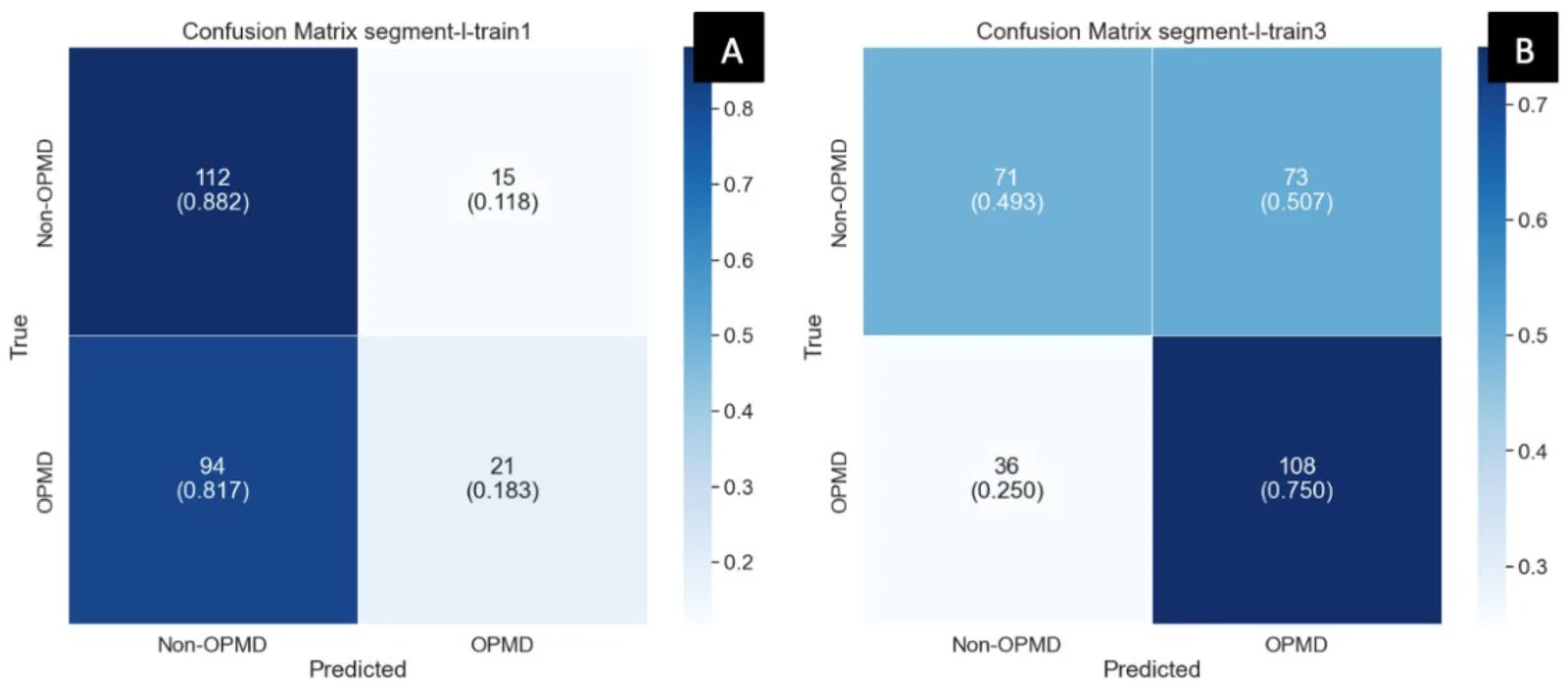
A 2 × 2 confusion matrix showing the binary classification results for the YOLOv8-large-segmentation model in Training A (**A**) and Training B (**B**). Diagonal values show the number of true positive (TP) and true negative (TN) predictions, and off-diagonal values show the number of false positive (FP) and false negative (FN) predictions for each class.

A total of 300 clinical test images (from Batch 1 to Batch 6) were used for model testing, with two testing runs performed following Training A and Training B respectively. Based on the YOLOv8 model’s prediction, there were four possible outcomes, namely true positives (TPs), true negatives (TNs), false positives (FPs), and false negatives (FNs). These outcomes were tabulated in a 2 × 2 confusion matrix, as shown in Figure 3, which formed the basis for calculating evaluation metrics. Accuracy was calculated as the proportion of correct predictions among all predictions. Sensitivity was calculated as TPs divided by the sum of TPs and FNs. Specificity was calculated as TNs divided by the sum of TNs and FPs. Precision was calculated as TPs divided by the sum of TPs and FPs. The F1 score was calculated as the harmonic mean of precision and sensitivity. These are image-level detection and classification metrics that quantify whether each photograph was correctly flagged as OPMD or non-OPMD; they do not measure pixel-level segmentation overlap such as the Dice similarity coefficient or Intersection over Union, which were not computed (see Section 4.5). Wilson 95% confidence intervals were derived from the confusion matrix cell counts for each metric.

## 3. Results

### 3.1. DenseNet-121 Classification Results

The DenseNet-121 model was assessed on the validation set across three training configurations. The results from each of the three training runs at 100 epochs, together with the training and validation losses as a function of epochs, are summarized in Table 2. At the end of 100 epochs, Training C was the only configuration in which training and validation losses had converged, which suggests adequate generalization. Training configurations B and C, however, required the manual cropping of every image to isolate the lesion. In detail, Training A reached a validation accuracy of 47.5% (training loss 0.64, validation loss 1.60) and Training B 56.5% (training loss 0.60, validation loss 1.70), with neither configuration converging, whereas Training C achieved 74.0% validation accuracy with closely aligned training and validation losses (0.44 and 0.57, respectively), being the only run to converge (Table 2).

This dependence on human pre-processing negates the automation that motivates AI-assisted screening, because a model that needs a human to first locate and crop the lesion offers little practical advantage over the human assessment it is meant to support. This finding is itself informative. It shows that a global image classifier, which integrates features across the whole photograph, is poorly matched to a task in which the diagnostic signal is spatially localized and surrounded by irrelevant structures such as teeth, mucosa, and instruments. This limitation directly motivated the adoption of a localization-aware, one-stage segmentation approach, which is reported below. DenseNet-121 was assessed by convergence behavior and validation accuracy only, whereas the YOLOv8 model was assessed on a held-out test set with full diagnostic metrics. The two models were therefore not evaluated on the same footing, and a direct numerical comparison between them was not undertaken.

### 3.2. Annotation Quality

Annotation validation rates were high and broadly consistent across annotators. Annotators 1, 2, and 3 achieved an average validation rate of approximately 97%, 93%, and 96% respectively. These high validation rates indicate that the training dataset was annotated to a consistent standard. These are first-round specialist validation (concordance) rates rather than chance-corrected inter-observer agreement: the three annotators worked on disjoint sets of images, and each annotation was validated by a single specialist, so no commonly rated subset exists on which Cohen’s or Fleiss’s kappa could be computed. Across the six batches, the overall first-round concordance was 95.8%.

### 3.3. YOLOv8-Large-Segmentation Results

Performance metrics for both training runs, with Wilson 95% confidence intervals, are summarized in Table 3. Expanding the training set markedly improved sensitivity, from 18.3% (95% CI 12.3 to 26.3) in Training A to 75.0% (95% CI 67.3 to 81.4) in Training B, with a corresponding rise in the F1 score from 27.8% to 66.5%. This gain in sensitivity was accompanied by a fall in specificity, from 88.2% (95% CI 81.4 to 92.7) to 49.3% (95% CI 41.3 to 57.4). The Training B specificity interval includes 50%, which indicates that the ability of the model to correctly identify non-OPMD cases could not be distinguished from chance at this test set size. Overall accuracy improved modestly to 62.2% (95% CI 56.4 to 67.6). Because the dataset was partitioned at the image level rather than the patient level, these test set metrics may be optimistically biased; this limitation is the central constraint on their interpretation and is examined in detail in Section 4.5.

Confusion matrix denominators were 242 and 288 for Trainings A and B respectively, which reflect the test images on which the model produced no prediction (the 4% non-detection noted above). These cases were excluded from metric calculation rather than counted as false negatives. The model produced color-coded instance segmentation masks (red for OPMD and blue for non-OPMD) that supported interpretable lesion localization. The examination of the misclassified cases showed that false positives arose mainly from non-OPMD lesions that clinically mimic OPMD (OSCC, frictional hyperkeratosis, morsicatio buccarum, and chronic hyperplastic candidiasis), whereas false negatives were predominantly erythroplakia and oral lichen planus; these overlapping presentations are the principal source of the reduced specificity.

This study performed a sensitivity analysis to test how this exclusion affected the reported figures. Treating the undetected images as false negatives reduces Training B’s sensitivity from 75.0% to between 69.2% (if every undetected image had contained OPMD) and approximately 72.0% (under a balanced assumption), with specificity essentially unchanged. The reported Training B sensitivity is therefore moderately robust to this accounting choice. The effect on Training A is larger, with sensitivity falling from 18.3% to as low as 12.1%, because a far greater proportion of images were undetected in this run. The exact breakdown of the undetected images by class was not recorded, so only these bounding scenarios can be reported.

## 4. Discussion

### 4.1. Model Selection: DenseNet-121 Versus YOLOv8

The one-stage detection and segmentation model, YOLOv8-large-segmentation, was selected over the classification-based model, DenseNet-121, because of its feature processing capabilities for localized tasks. DenseNet-121 operates as a global image classifier that analyzes entire images and provides classifications, whereas the YOLOv8-large-segmentation model targets the lesion areas through bounding boxes and segmentation masks and effectively excludes irrelevant features such as teeth, the tongue, and dental instruments. The model generates color-coded masks (red for OPMD, blue for non-OPMD) that delineate the regions of interest.

The preliminary analysis showed that the DenseNet-121 architecture was not suitable for a study involving localized features. Extensive manual image cropping to focus only on lesions negated the intended automation benefits of AI-assisted OPMD detection. Substantial human input was needed to direct the model towards specific lesion features rather than surrounding areas, which led to an impractical workflow. In contrast, the segmentation-based YOLOv8-large model was a more suitable choice for a task that required accurate lesion detection and segmentation. This is consistent with findings by Tanriver et al. [20] and Keser et al. [21,32], who reported the advantage of detection-based architectures over global classifiers for oral lesion tasks.

This conclusion is deliberately narrow. DenseNet-121 is not a state-of-the-art classifier, and a modern Vision Transformer or Swin Transformer could outperform it on the same whole-image classification task because of stronger global attention and richer feature extraction. We therefore do not claim that segmentation architectures are intrinsically superior to classification architectures. What our data support is that a whole-image classification framing was poorly matched to this localized task, because it needed manual lesion cropping to converge and did not provide the lesion-level localization and interpretable masks that the clinical use case requires. This limitation arises from the whole-image framing rather than from the age of the DenseNet backbone, and it would apply, though perhaps to a lesser degree, to a transformer-based whole-image classifier. A direct comparison of detection and segmentation models against modern transformer classifiers, under matched data and evaluation protocols, is an important direction for future work.

### 4.2. YOLOv8 Performance and Clinical Relevance

The YOLOv8-large-segmentation model achieved a sensitivity of 75.0% when trained on the full 1200-image dataset. This figure should be read alongside its 95% confidence interval (67.3 to 81.4) and the low confidence threshold at which it was obtained, rather than compared directly with published figures for unaided general dental practitioner OPMD screening (sensitivity 57.8%) [11]. Sensitivity is an important metric in this context, because false negatives, that is missed OPMD, carry a high clinical risk by delaying intervention. A direct performance comparison between DenseNet-121 and YOLOv8 was not undertaken, given the fundamental differences in output metrics, training dataset sizes, and task types.

The observed specificity of 49.3% reflects a high false positive rate, which is partly attributable to the low confidence threshold of 0.1 and to the visual overlap between non-OPMD lesions (for example OSCC, frictional hyperkeratosis, morsicatio buccarum, and chronic hyperplastic candidiasis) and true OPMD. The confidence interval for this estimate includes 50%, so the result should be read as performance that cannot be distinguished from chance for the non-OPMD class at this test set size. Future iterations should explore systematic threshold optimization using ROC curve analysis [21].

These findings should be read against the published benchmarks rather than equated with them. Recent meta-analyses report higher pooled figures, with one 2024 review reporting pooled sensitivity near 0.87 and specificity near 0.81, although this pooled specificity is driven substantially by histopathological imaging rather than by white-light clinical photographs [33]. A 2025 meta-analysis restricted to clinical image studies reported a lower pooled specificity of 0.67 (95% CI 0.58 to 0.75), with that for individual studies ranging from 0.58 to 0.79 [34]. Our specificity of 49.3% is below even the lower bound of the photograph-based pooled estimates, and we do not present it as competitive. We present it as the finding of a small-dataset feasibility study, and the gap relative to these benchmarks reflects the dataset scale, curation, and validation that the field standard requires and that our study identifies as the path forward.

We use the term feasibility in its methodological sense. This study does not demonstrate a clinically ready usable tool yet, and the observed specificity of 49.3%, whose confidence interval includes 50%, shows that the present configuration cannot reliably distinguish non-OPMD cases. What this study establishes is narrower. White-light photographs carry sufficient signal for a localization-aware model to detect candidate lesions in 96% of test images, the segmentation framing overcomes the manual cropping barrier that made the classification approach impractical, and the specific cause of the low specificity is identified together with a defined remediation path through threshold optimization, larger and patient-stratified data, and a multi-class scheme. On this basis, we argue that the approach merits further development, not that it is ready for use.

False negative cases were predominantly erythroplakia and OLP, driven by visual similarity to red non-OPMD lesions and by the morphological heterogeneity of OLP subtypes respectively.

### 4.3. Annotation Quality

The accuracy of segmentation mask outputs is tied to the quality of the annotated training dataset. To reduce annotation bias, annotation tasks were distributed evenly among three designated researchers, with all annotations validated by three other researchers with expertise in Oral Pathology and Medicine. This is consistent with accepted practice for medical image annotation [35]. Validation rates of 93% to 97% support training data quality, although they reflect specialist validation rather than formal inter-annotator agreement statistics. Anantharaman et al. [36] showed the viability of instance segmentation for oral lesion differentiation, which supports the feasibility of this approach.

### 4.4. Clinical Implications

The detection of OPMD from standard clinical photographs that can be captured with consumer-grade smartphones opens a possible pathway to low-cost, non-invasive triage for lay users performing self-examination and for general dental practitioners with limited OPMD training [15]. Any such tool must be positioned explicitly as an adjunct to specialist clinical assessment and histopathological biopsy, which remains the gold standard [14,37]. General practitioners could in principle use a mature version of this approach to support provisional diagnosis and the prioritization of specialist referrals, particularly in regions where specialist appointments are delayed or inaccessible. The performance reported here does not yet support such use, and the implications described are prospective rather than demonstrated.

### 4.5. Limitations

Several limitations constrain the interpretation of these findings. The most important concerns data partitioning. The dataset was split at the image level rather than the patient level. For images from the institutional archive, more than one image of the same patient or lesion may exist, and for images obtained from public online sources, patient identity is not recoverable. We were therefore unable to guarantee patient-level separation between the training and test partitions, and we cannot exclude the possibility that correlated images of the same patient or lesion were present on both sides of the split. Where such leakage exists, the reported test set metrics would be optimistic by an amount we cannot quantify from the available records. A patient-stratified re-evaluation was not possible, because this study has concluded and the necessary patient-level identifiers are not available for the public-source images. We regard this as the central constraint on the interpretation of our results, and prospective patient-level partitioning is an essential requirement for any follow-up study.

Further, the dataset was partly assembled from publicly accessible online sources, and despite strict manual curation, we cannot fully exclude accidental near-duplicate images, so future work should apply automated perceptual de-duplication before splitting. Performance metrics are reported as point estimates with Wilson 95% confidence intervals derived from a 300-image test set. The resulting intervals are wide. The Training B specificity interval (41.3% to 57.4%) includes 50% and so indicates performance indistinguishable from chance for the non-OPMD class, and this study was not powered to the 90% sensitivity and specificity targets assumed in the a priori sample size calculation. The reported sensitivity was obtained at the deliberately low confidence threshold of 0.1 chosen to favor detection, which inflates sensitivity at the direct expense of specificity, and no formal threshold optimization, such as ROC curve analysis, was performed. Images on which the model produced no prediction (4% of the test set) were excluded from the confusion matrix rather than counted as missed detections, which modestly inflates the reported sensitivity, as quantified in the sensitivity analysis in Section 3.3.

Standardized object detection and segmentation metrics were not computed. Mean average precision at IoU 0.5 (mAP@0.5, equivalent to AP50 for a single class) and averaged across 0.5 to 0.95 (mAP@0.5:0.95), together with the Dice similarity coefficient, require the per-image prediction confidences and the saved per-image predicted masks, none of which were retained, and they cannot be recovered from a thresholded confusion matrix that represents a single operating point. As a result, the spatial accuracy of the segmentation masks was assessed only qualitatively for interpretability and was never measured quantitatively. The masks should therefore be read as spatially indicative rather than as validated for accuracy. Both mAP and the Dice coefficient should be reported in any follow-up study, since they are expected standards for detection and segmentation work.

OSCC was deliberately included within the non-OPMD class as a differential diagnosis. OSCC is, by definition, a malignancy and not a potentially malignant disorder, so it could not be placed in the OPMD class, and it was retained in the dataset rather than excluded because it is a clinically important visual differential of several OPMDs, and its exclusion would have produced a dataset that did not reflect real screening conditions. We accept the clinical danger this creates. A binary scheme that places OSCC in the negative class can assign a frank malignancy to the lower-concern category, which is the most serious possible failure for a triage tool. For this reason, we recommend a multi-class scheme separating malignant, potentially malignant, and benign lesions as a priority for future work. The model also had no access to patient-level metadata such as demographics, risk habits, and lesion duration, which specialists routinely use to resolve diagnostic uncertainty [38]. This study is retrospective and single-source for the institutional component, and external validation on independent, prospectively collected, multicenter datasets is required before any clinical application can be considered.

### 4.6. Future Directions

The dataset of 1500 images is modest compared with high-performing deep learning systems. Dataset expansion is a priority, especially for rare conditions such as erythroplakia. Targeted collection through outreach in high-OPMD-prevalence regions would enrich the training data and could serve as a community oral cancer screening initiative. Future multimodal architectures that integrate structured questionnaire data with image features, as demonstrated in dermatology [39], should be explored. The binary OPMD or non-OPMD scheme should be refined to distinguish OSCC from benign non-OPMD conditions. Future work should also benchmark detection and segmentation models against modern transformer-based classifiers, such as Vision Transformer and Swin Transformer variants, under matched data and evaluation protocols, so that the relative contribution of task framing and the backbone architecture can be separated. External validation on independent multicenter datasets is required before clinical deployment.

## 5. Conclusions

This proof-of-concept study establishes that one-stage instance segmentation is a viable and clinically interpretable architectural direction for detecting OPMD from standard white-light intraoral photographs and that it can generate spatially interpretable segmentation masks suitable for clinical communication, whereas whole-image classification framing was poorly matched to this localized task. At the present dataset scale of 1200 training images, however, performance, and particularly a specificity of 49.3%, is not sufficient for clinical deployment, and the encouraging sensitivity of 75.0% was obtained at a permissive confidence threshold that must be formally optimized in future work.

The contribution of this study lies less in the performance figures themselves than in the specific, quantified barriers it identifies, namely insufficient dataset scale, uncalibrated decision thresholds, the absence of patient-level metadata, and the visual overlap between OPMD and several benign mimics. Addressing these, through dataset expansion, ROC-based threshold optimization, the reporting of standardized detection metrics such as mAP, the multimodal integration of clinical metadata, and external multicenter validation, constitutes a concrete and tractable research agenda. White-light photograph-based OPMD triage is not yet clinically ready, but this work shows that it is a direction worth pursuing and defines what future researchers should do.

## Figures and Tables

**Table 1:** The oral lesions and conditions included in this study and the number of images for each entity.

Class	Condition	No. of Images
OPMD	Erythroplakia	125
	Leukoplakia	125
	Proliferative verrucous leukoplakia (PVL)	125
	Oral submucous fibrosis (OSMF)	125
	Oral lichenoid lesion (OLL)	125
	Oral lichen planus (OLP)	125
Non-OPMD	Oral squamous cell carcinoma (OSCC)	54
	Leukoedema	36
	White sponge nevus	36
	Frictional hyperkeratosis	42
	Morsicatio mucosa, buccarum, linguarum	36
	Chemical burn	36
	Oral candidiasis (chronic hyperplastic)	48
	Oral candidiasis (erythematous)	48
	Oral candidiasis (acute pseudomembranous)	48
	Oral hairy leukoplakia	36
	Benign migratory glossitis	36

**Table 2:** The image classification results from the three training runs of the DenseNet-121 model at 100 epochs.

Evaluation Metric	Training A	Training B	Training C
Training loss	0.64	0.60	0.44
Validation loss	1.60	1.70	0.57
Validation accuracy	0.475	0.565	0.740

**Table 3:** YOLOv8-large-segmentation model performance metrics for Trainings A and B, with Wilson 95% confidence intervals. Denominators reflect images on which the model produced a prediction (242 for Training A and 288 for Training B). Images with no detection were not included in the matrix.

Evaluation Metric	Training A	95% CI	Training B	95% CI
Accuracy (%)	55.0	48.7 to 61.1	62.2	56.4 to 67.6
Sensitivity or Recall (%)	18.3	12.3 to 26.3	75.0	67.3 to 81.4
Specificity (%)	88.2	81.4 to 92.7	49.3	41.3 to 57.4
Precision (%)	58.3	42.2 to 72.9	59.7	52.4 to 66.5
F1 Score (%)	27.8	Not applicable	66.5	Not applicable

## Data Availability

The data presented in this study are available on request from the corresponding author. Publicly sourced images were obtained from openly accessible online sources; institutional images are not publicly available owing to patient privacy restrictions.
